# Characterization of IXINITY® (Trenonacog Alfa), a Recombinant Factor IX with Primary Sequence Corresponding to the Threonine-148 Polymorph

**DOI:** 10.1155/2016/7678901

**Published:** 2016-02-21

**Authors:** Dougald M. Monroe, Richard J. Jenny, Kevin E. Van Cott, Shelly Buhay, Laura L. Saward

**Affiliations:** ^1^School of Medicine, Department of Hematology/Oncology, University of North Carolina at Chapel Hill, Chapel Hill, NC 27599, USA; ^2^Haematologic Technologies, Incorporated, Essex Junction, VT 05452, USA; ^3^Department of Chemical and Biomolecular Engineering, University of Nebraska-Lincoln, Lincoln, NE 68588, USA; ^4^Biosciences Division, Emergent BioSolutions Incorporated, Winnipeg, MB, Canada R3T 5Y3; ^5^University of Manitoba, Winnipeg, MB, Canada R3T 2N2

## Abstract

The goal of these studies was to extensively characterize the first recombinant FIX therapeutic corresponding to the threonine-148 (Thr-148) polymorph, IXINITY (trenonacog alfa [coagulation factor IX (recombinant)]). Gel electrophoresis, circular dichroism, and gel filtration were used to determine purity and confirm structure. Chromatographic and mass spectrometry techniques were used to identify and quantify posttranslational modifications. Activity was assessed as the ability to activate factor X (FX) both with and without factor VIIIa (FVIIIa) and in a standard clotting assay. All results were consistent across multiple lots. Trenonacog alfa migrated as a single band on Coomassie-stained gels; activity assays were normal and showed <0.002 IU of activated factor IX (FIXa) per IU of FIX. The molecule has >97%  *γ*-carboxylation and underwent the appropriate structural change upon binding calcium ions. Trenonacog alfa was activated normally with factor XIa (FXIa); once activated it bound to FVIIIa and FXa. When activated to FIXa, it was inhibited efficiently by antithrombin. Glycosylation patterns were similar to plasma-derived FIX with sialic acid content consistent with the literature reports of good pharmacokinetic performance. These studies have shown that trenonacog alfa is a highly pure product with a primary sequence and posttranslational modifications consistent with the common Thr-148 polymorphism of plasma-derived FIX.

## 1. Introduction

Human factor IX (FIX) is a vitamin K-dependent glycoprotein that circulates in plasma at a concentration of approximately 4 *μ*g/mL. It is a 415 amino acid protein that is synthesized in the liver and contains multiple posttranslational modifications (PTMs), including O-glycosylation, N-glycosylation, *γ*-carboxylation, sulfation, and phosphorylation [1–10]. FIX circulates in plasma as a zymogen that is proteolytically activated to an enzyme, FIXa, during the process of blood coagulation. The activation of FIX by FXIa occurs on the platelet surface [11] and the resulting FIXa acts on the platelet surface as part of a factor Xa- (FXa-) generating enzyme complex [12]. FIXa alone has minimal ability to activate factor X (FX), but when assembled into a metal ion-dependent complex with the cofactor, factor VIIIa (FVIIIa), the complex efficiently activates FX to FXa. [13]. In a similar manner, the resulting FXa forms an enzyme complex with factor Va (FVa) that proteolytically activates prothrombin to provide the burst of thrombin generation necessary for fibrin clot formation [12]. The overall importance of FIX is indicated by the fact that a deficiency of FIX results in a severe bleeding disorder, hemophilia B [14].

The preferred method of management of bleeding in hemophilia B patients is administration of plasma-derived or recombinant FIX products. While recombinant FIX products are similar to the natural protein, none are identical in every detail. Various posttranslational modifications are the primary reason for such differences, but also of interest is a polymorphism found in plasma-derived FIX [15]. The polymorphism occurs at residue 148 with two identified variants: Thr-148 and Ala-148. The relative proportion of this polymorphism varies among populations, although Thr-148 is always predominant [16]. To date, recombinant FIX products have all been developed to be consistent with the Ala-148 variant. In contrast, IXINITY (trenonacog alfa [coagulation factor IX (recombinant)]) is a new, FDA-licensed recombinant human FIX with a primary amino acid sequence having the predominant Thr-148 polymorphism.

Trenonacog alfa is produced in a Chinese Hamster Ovary (CHO) cell line. The purification process is comprised of chromatographic purification steps and three validated viral inactivation/removal steps, which include solvent-detergent treatment, a chromatographic step, and nanofiltration. The purification process also contains a validated polishing step for the reduction of CHO proteins.

In this study, trenonacog alfa was extensively characterized using a number of analytical techniques to assess primary structure, higher order structure, function, and purity.

## 2. Methods

### 2.1. One-Dimensional SDS-PAGE

SDS-PAGE was performed using NuPAGE® 12% or 4–12% gradient acrylamide gels and 4x LDS sample buffer from Life Technologies (Grand Island, NY). Samples were reduced with *β*-mercaptoethanol (5% v/v final). Protein loads were 2 *μ*g/lane for standard Coomassie Blue stained gels and 1.2 *μ*g/lane for kinetic experiments (also stained with Coomassie Blue). The MW marker was SeeBlue® Plus 2 standard (Life Technologies). Gels were run at 175 or 200 V for 68 or 53 minutes, respectively, in a MOPS-buffered SDS running buffer (Life Technologies).

### 2.2. Gel Filtration Chromatography

GFC was performed using a TSKgel® G3000SWXL column (7.8 mm × 30 cm; 5 *μ*m particle size; Tosoh Bioscience (King of Prussia, PA)) on an Agilent 1100/1200 HPLC system (Santa Clara, CA) held at 22°C. Chromatography was performed at a flow rate of 0.5 mL min^−1^, with a mobile phase of 50 mM Tris, 200 mM NaCl, pH 7.5, in the presence and absence of 5 mM CaCl_2_. UV data were collected at 280 nm. A 40 *μ*g protein load was used.

### 2.3. Reduced Reverse Phase HPLC

Samples were simultaneously denatured and reduced using guanidine hydrochloride and dithiothreitol (6 M and 10 mM final concentration, resp.; 30 min at room temperature (RT)). Next, samples were alkylated using 4-vinylpyridine (3 mM final concentration; 90 min at RT, protected from light). Chromatographic separation was performed using a Zorbax 300SB-C18 column (4.6 mm × 100 mm; 3.5 *μ*m) installed on an Agilent 1100/1200 HPLC system in gradient mode. The starting mobile phase was 95%  A and 5%  B, where “A” is 0.1% (v/v) trifluoroacetic acid (TFA) in water and “B” is 0.1%  TFA in 25% isopropyl alcohol, 75% acetonitrile. A nonlinear gradient from 5%  B to 52%  B was performed over 67 minutes, followed by a wash step with 100%  B. The flow rate was 1 mL min^−1^, and the column was maintained at 30°C. A 25 *μ*g protein load was used.

### 2.4. Host Cell Protein ELISA

Host cell proteins were quantified by a sandwich ELISA that incorporates the use of an affinity-purified, sheep anti-host cell protein polyclonal antibody for both capture and detection. The sheep antibody was produced by immunization with a preparation of host cell proteins derived from a mock (null vector) factor IX production run (Hematologic Technologies Incorporated, Essex Junction, VT). Samples or standards and the horseradish peroxidase- (HRP-) conjugated detecting antibody were simultaneously dispensed onto assay plates passively coated with capture antibody and were allowed to bind. The plate was then washed and the HRP substrate 3,3,5′,5′-tetramethylbenzidine (Sigma-Aldrich, St. Louis, MO) was added to the plate. Following a short development step, the reaction was quenched with sulfuric acid and the plate was read at 450 nm. Absorbance was converted to concentration using assay calibration standards that were also generated from a null vector run.

### 2.5. Nonactivated Partial Thromboplastin Time

Nonactivated partial thromboplastin times (NAPTTs) were measured by European Pharmacopoeia (Ph. Eur.) method 2.6.22, Activated Coagulation Factors. Pass/fail was based on a threshold clotting time of 150 s.

### 2.6. Chromogenic Assay for Residual FIXa

The BIOPHEN® Factor IXa assay kit (Aniara Diagnostica, West Chester, OH) was used in accordance with the manufacturer's instructions to quantify trace FIXa present in trenonacog alfa.

### 2.7. N-Terminal Sequencing

N-terminal sequencing was performed by Edman degradation using a Procise® Sequencer (Applied Biosystems, Carlsbad, CA).

### 2.8. Peptide Mapping

LC-MS/MS peptide mapping was performed to confirm primary sequence and to identify and quantitate PTMs. A LysC (Achro K, Wako, Osaka, Japan) digest was performed for general sequence coverage. A PNGase F plus trypsin digest was used to specifically analyze the O-glycosylation, phosphorylation, and sulfation modifications of the activation peptide. For LysC digests, samples were diluted in 50 mM ammonium bicarbonate buffer (pH 7.8), reduced with dithiothreitol (5 mM final), alkylated with iodoacetamide (15 mM final), and then digested with LysC (1 : 50 E : S, w : w) for 18 hours at 30°C. For trypsin digests, samples were deglycosylated with PNGase F (New England Biolabs, Ipswich, MA) using 0.5 U *μ*g^−1^ of FIX for 2 hours at 37°C, reduced and alkylated as described above, and then digested with trypsin (Promega; Fitchburg, WI; 1 : 50 E : S, w : w) for 18 hours at 37°C. Digests were quenched to a final 0.5% formic acid concentration, aliquoted, and then frozen at −80°C until LC-MS/MS analysis.

LC-MS/MS analysis was performed on a Dionex*™* U3000 nanoLC system (Sunnyvale, CA) with an AB-Sciex 4000 QTrap® MS (Framingham, MA). Solvent A was 0.1% formic acid (v/v) in deionized water. Solvent B was 0.1% formic acid (v/v) in acetonitrile (Burdick and Jackson, Muskegon, MI). Peptides were separated on a Dionex Acclaim Pepmap100 C18 column (75 *μ*m × 25 cm, 3 *μ*m particle size) with an Acclaim Pepmap100 C18 trap column used to desalt and concentrate the samples prior to separation (200 *μ*m × 1 cm, 5 *μ*m particle size). The columns were preequilibrated with 100%  A and maintained at 45°C. After injection onto the trap column and a 100%  A wash, the valve was switched to separation mode, and a linear gradient to 5%  B over 5 minutes followed by a linear gradient to 45%  B over 200 minutes (250 nL min^−1^) was used to separate the peptides. MS data were acquired in positive mode, with collision energies optimized to maximize the information content of the MS/MS spectra.

### 2.9. Circular Dichroism Spectroscopy

Trenonacog alfa samples (2.5 mg mL^−1^) were treated with 0.1 mg/mL Chelex® 100 (Bio-Rad, Hercules, CA) for approximately fifteen minutes with gentle agitation and then centrifuged and filtered. Samples were then diafiltered and brought to a final concentration of 1.0 mg/mL in 25 mM MOPS, 150 mM NaClO_4_, 5 mM CaCl_2_, and pH 7.5. CD measurements were carried out using a Jasco J-715 Spectropolarimeter (Easton, MD).

### 2.10. *γ*-Carboxylation

*γ*-Carboxylation was quantified by strong anion exchange chromatography, as per Gillis et al. with a gradient optimized for the chromatography system and a decreased flow rate (0.5 mL min^−1^) [17].

### 2.11. FIX Clotting Assay

A FIX one-stage clotting assay was performed on an ACL-TOP coagulation analyzer (Beckman Coulter, Brea, CA). The aPTT reagent (HemosIL Synthasil), FIX deficient plasma, and diluent were from Instrumentation Laboratories (Bedford, MA). The assay was calibrated against the British Working Standard for factors II, IX, and X (NIBSC Catalog #07/326). Data were analyzed by the parallel line method using PLA 2.0 software (Stegmann Systems, Rodgau, Germany).

### 2.12. Rate of Activation by Factor XIa

The rate of FIX activation by FXIa was determined using SDS-PAGE to monitor the disappearance of the 55 kDa FIX band and the appearance of the 45 kDa FIXa band in a time-course experiment. FXIa was incubated with FIX at a mass ratio of 1 : 1,000, and samples were withdrawn at 5-minute intervals and run on nonreducing SDS-PAGE gels with Coomassie staining. Optical densities of the FIX and FIXa bands in each sample were calculated as a proportion of the total optical density of the lane. The relative proportions were plotted as a function of time and fit by linear regression, where the slope equaled the rate of conversion of FIX to FIXa.

### 2.13. Effect of Cofactor (FVIIIa) on FIXa Activity

The enhancement of FIXa activity by FVIIIa was examined using the Biophen Factor IX assay kit (Aniara Diagnostica, West Chester, OH) in two separate trials: one where FVIIIa was included in the assay reagent, and another where FVIIIa was not included. Samples were assayed at two or more different concentrations such that reaction rates could be calculated by plotting assay results against FIXa concentration. The ratio of slopes (rate with FVIIIa/rate without FVIIIa) was employed to express the relative rate enhancement with the addition of FVIIIa.

### 2.14. Conversion of FIX to FIXa

Several methods required the complete conversion of FIX to FIXa. This was accomplished by incubating FIX with FXIa at an enzyme to substrate ratio of 1 : 100 (w/w) for 40 minutes at 37°C. SDS-PAGE was employed to confirm complete activation.

#### 2.14.1. Inhibition of FIXa by Antithrombin

The efficiency of antithrombin inhibition was determined by incubating 1.09 *μ*M FIXa with varying concentrations of antithrombin (0 to 1.4 *μ*M). The residual FIXa activity in each of the samples was then measured using the Biophen FIXa assay kit (Aniara Diagnostica, West Chester, OH). Residual FIXa activity was plotted as a function of the antithrombin concentration and data were then analyzed by linear regression.

### 2.15. Active-Site Titration

The number of active sites per FIXa molecule was determined using the synthetic “burst” reagent 4-nitrophenyl 4-guanidinobenzoate hydrochloride in accordance with published methods [18].

### 2.16. Sialic Acid Quantitation

The N-acetylneuraminic acid (Neu5Ac) content of trenonacog alfa was quantitatively determined by the method of Anumula [19]. Samples and standards were analyzed with triplicate mild acid hydrolyses. Neu5Ac calibration standards went through the hydrolysis reaction in parallel with the samples. The released sialic acids were labeled with o-phenylenediamine (OPD) and then separated by RP-HPLC (duplicate injections of each labeled hydrolysate). A linear calibration curve was used to calculate the total amount of Neu5Ac per mole of FIX.

### 2.17. N-Glycan Profile

N-glycan profiling was performed according to the method described by Anumula and Dhume [20]. Glycan populations were analyzed by MALDI-TOF-MS to determine sialylation levels in each region of the normal phase chromatogram.

## 3. Results

### 3.1. Purity

**Figure 1:**
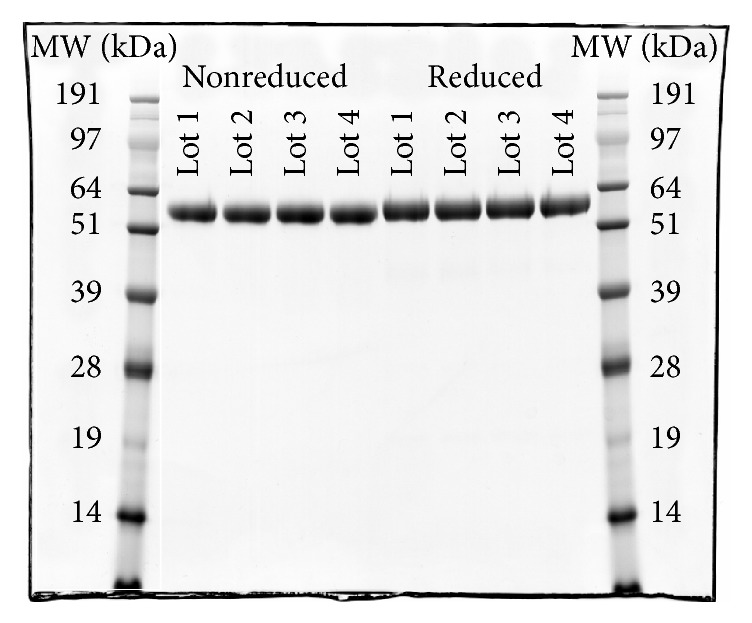
SDS-PAGE was performed using a NuPAGE 12% gel and 4x LDS sample buffer from Life Technologies (Grand Island, NY). Reduced lanes were treated with *β*-mercaptoethanol to 5% of total sample volume. 2 *μ*g of protein was loaded in each lane. Gels were stained with Coomassie Blue. Lanes are (from left to right), SeeBlue Plus 2 standard, lot 1, lot 2, lot 3, lot 4, lot 1 reduced, lot 2 reduced, lot 3 reduced, lot 4 reduced, and SeeBlue Plus 2 standard. Molecular weights corresponding to the standards are shown.

**Figure 2:**
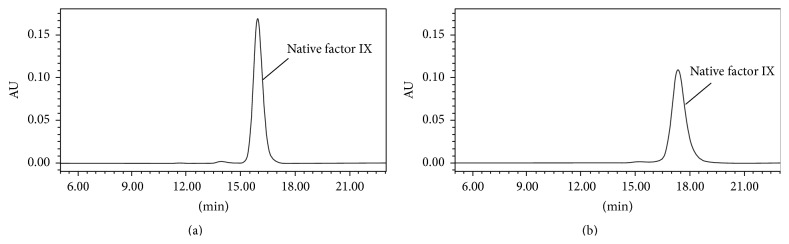
Gel filtration chromatography was performed using a TSK-Gel G3000SWXL column run at 22°C at a flow rate of 0.5 mL min^−1^. The column was run in 50 mM Tris, 200 mM NaCl, and pH 7.5. 40 *μ*g of total protein was loaded on the column per run. Absorbance at 280 nm is plotted against time in minutes. The upper tracing shows trenonacog alfa run in the absence of calcium. The lower tracing shows trenonacog alfa run in the presence of 5 mM CaCl_2_.

Purity of trenonacog alfa was assessed using a panel of assays designed to detect high molecular weight (HMW) species and proteolytic degradants. By SDS-PAGE with Coomassie stain, under both reducing and nonreducing conditions, only a single band at apparent molecular weight 57,000 Da was detected for each trenonacog alfa lot tested, indicating that the dominant species in the preparations was full length, unactivated FIX (Figure 1). The apparent molecular weight is consistent with the mature plasma-derived protein, complete with the expected posttranslational modifications. Trenonacog alfa was further analyzed by gel filtration chromatography and high molecular weight species were found to be present at very low levels (≤1.3%) (Figure 2(a)). The presence of these species was confirmed by analytical ultracentrifugation (data not shown). Trenonacog alfa samples were also reduced and alkylated and then subjected to reversed-phase HPLC analysis, which permitted proteolytic degradants to be separated and quantified. Total levels of proteolytic degradants were found to be ≤6%, where FIXa light chain and heavy chain associated species made up the majority of the detected material (approximately 2% and 3%, resp.). A truncated form of FIX (cleaved at C-terminus of Arg318) made up the remaining approximately 1%. This form has been previously reported in recombinant FIX products [21].

Residual proteins from the expression host (Chinese Hamster Ovary cells) were detected by sandwich ELISA, using capture and detection antibodies raised against partially purified lysate from a null vector manufacturing run. This method was developed to be highly sensitive to host cell proteins that copurify in the trenonacog alfa manufacturing process. Using this ELISA, it was found that early development lots (identified as IB1001) had detectable levels of host cell protein. A change in the manufacturing process was introduced for the current product (IXINITY) such that the host cell protein levels were reduced a thousandfold.

Two assays were used to evaluate FIXa levels in trenonacog alfa. The nonactivated partial thromboplastin time assay (NAPTT), which is a universal test for the presence of activated coagulation factors, yielded consistent results on all lots tested and indicated only minimal contamination with FIXa. Additionally, a commercially available, kit-based colorimetric assay specific for FIXa was used to quantify FIXa levels in seven lots of trenonacog alfa; measured values were consistent from lot-to-lot and in the range of ≤0.0019 IU FIXa/IU FIX.

### 3.2. Primary Structure and Posttranslational Modifications

The primary structure for trenonacog alfa, including the threonine residue at position 148, was confirmed through peptide mapping (AchroK, trypsin) and N-terminal sequencing (first ten residues of the activation peptide). Mass spectrometry by MALDI-TOF indicated a weight average molecular weight of 54827 Da, which differs from the theoretical mass of the amino acid sequence due to the numerous PTMs [22]; a summary of the posttranslational modifications found in trenonacog alfa is given in Table 1.

### 3.3. Higher Order Structure

**Figure 3:**
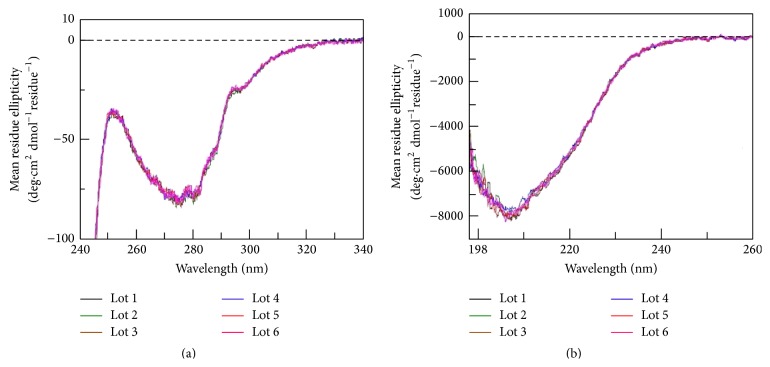
Circular dichroism spectroscopy was carried out in 25 mM MOPS, 150 mM NaClO_4_, 5 mM CaCl_2_, and pH 7.5 at approximately 1.0 mg mL^−1^ factor IX. CD measurements were carried out with a 1 cm cell for near UV measurements and a 0.02 cm cell for far UV measurements. Five accumulations were obtained per spectrum. The spectrum of the buffer blank was subtracted from the sample spectra; then the observed ellipticities were converted to mean residue ellipticities using the protein concentration, the mean residue weight (112.2 Da), and the path length of the cell. Near UV spectra are shown in the upper panel. Far UV spectra are shown in the lower panel. The spectra from six trenonacog alfa lots are shown on each plot.

Higher order structure was evaluated by circular dichroism (CD) spectroscopy in the presence of calcium ion; under these conditions, FIX is known to undergo a conformational change, resulting in a more ordered structure that is required for activity [23–25]. Both secondary and tertiary structures were highly consistent, as illustrated by the spectra obtained in both the near and far UV for six trenonacog alfa lots (see Figure 3). The conformational change was also verified by gel filtration chromatography, where native FIX in trenonacog alfa samples elutes later in the presence of calcium ion compared to its absence (see Figure 2), even though the molecular masses are equivalent by static light scattering (data not shown). The increased elution time can be attributed to the lower hydrodynamic volume of the more ordered conformation.

### 3.4. Functional Characterization

The functional properties of trenonacog alfa were evaluated by a selected panel of* in vitro* tests. An aPTT-based, single-stage, FIX clotting assay was employed to measure potency relative to an appropriate international standard for recombinant FIX. As illustrated in Table 2, the six lots of trenonacog alfa tested yielded a mean potency of 247 IU/mg with good overall lot-to-lot consistency (RSD = 7.15%). To supplement the potency data, a set of more discrete assays were employed to examine very specific properties of the molecule. The various properties examined included (a) the rate of activation by factor XIa; (b) the effect the cofactor FVIIIa on the FIXa activity; (c) the efficiency of inhibition by antithrombin; and (d) the number of active catalytic sites per molecule, once fully activated. The results obtained from these various assays are presented in Table 2, and in all cases, they yielded values that were (a) consistent from lot-to-lot and (b) within a range that would be expected for plasma-derived FIX.

## 4. Discussion

The standard of care for managing hemophilia B patients is replacement therapy using recombinant or plasma-derived FIX products. Although both recombinant and plasma-derived FIX products are viewed by most as equally safe and efficacious, there are subtle differences that serve to distinguish between products.

There is at least one prevalent polymorphism that has been identified in FIX. Residue 148, the third residue in the activation peptide, is either an alanine or a threonine (Malmö polymorphism) [16]. Threonine is more common at this position. Alanine is found in up to 35% of some European populations, much lower frequency (3–7%) in Asian populations, and is not seen at all in some African populations [16]. There does not seem to be any selective advantage to the mutation, and epitope mapping of inhibitors from hemophilia B patients has not identified patient antibodies that recognize this site. In plasma-derived FIX, the distribution of Ala/Thr would be expected to be representative of the population from whom the plasma is drawn. Recombinant products BeneFIX (nonacog alfa [coagulation factor IX (recombinant)]), which has been available for more than fifteen years, and Rixubis (nonacog gamma [coagulation factor IX (recombinant)]), a relatively new product licensed in 2013, both have alanine at residue 148. IXINITY (trenonacog alfa [coagulation factor IX (recombinant)]) has the more common threonine at residue 148.

Modern therapy is to give the highest purity product possible. Plasma-derived FIX preparations can, unsurprisingly, contain other Gla-containing proteins including FVIIa and FX, although these proteins were not detected in the highest purity plasma-derived products [3, 26]. Preparations of recombinant proteins do not contain other coagulation factors but may contain proteins native to the cell line producing the protein. Early in development, elevated levels of host cell proteins were reported in trenonacog alfa (which was identified in these reports as IB1001); this resulted in seroconversion in a subset of patients [27]. Modifications to the manufacturing process and the introduction of enhanced test methods have ensured adequate removal of these proteins in the current product. This is supported by clinical data, as no new seroconversions have been identified in patients receiving new product, and patients who had previously seroconverted with measurable titers and who received trenonacog alfa (IXINITY) have shown a decreasing trend in titer values [28].

In preparations of both plasma-derived and recombinant FIX, both higher and lower molecular weight contaminants have also been observed [3, 26]. The purity of trenonacog alfa in this study was consistent with previous reports for FIX therapeutics [1, 4, 25, 29, 30]. Small amounts of higher molecular weight products were present as were low levels of material consistent with peptides resulting from cleavage at Arg145 (carboxy terminal of light chain), Arg180 (amino terminal of heavy chain), and Arg318. All sites are known to be sensitive to cleavage by proteases [31].

The presence of light chain in this analysis suggests the possibility that some of the protein might have been activated during purification. The literature supports that there is a correlation between high FIXa levels and thrombogenicity in FIX preparations [29]. Residual FIXa has been identified as a contaminant in FIX preparations, both plasma-derived and recombinant [1, 4, 25, 29, 30]. Trenonacog alfa was evaluated for FIXa levels. A chromogenic assay was used to establish that levels of FIXa were low (≤0.0019 IU/IU FIX). The role of these levels in promoting clotting was examined by the standard European Pharmacopoeial method using nonactivated partial thromboplastin times [32]. This assay provides a threshold clotting time; clotting times shorter than the threshold are regarded as being indicative of a relevant enhancement of procoagulant activity. All samples had clotting times longer than the threshold indicating that the activity of the FIXa in these trenonacog alfa lots was below the levels associated with thrombosis [33].

FIX undergoes extensive posttranslational modification. In FIX, like the other vitamin K-dependent coagulation proteins, a number of glutamic acid (Glu) residues in the amino-terminal end of the molecule undergo modification by addition of an extra carboxyl group. The addition of this carboxyl group produces gamma-carboxyglutamate (Gla) [34]. In native human FIX, there are 12 such Gla residues [2]. This modification is necessary for calcium ion binding which significantly alters the structure of FIX, enabling binding to a negatively charged lipid surface [35]. Analysis of the Gla content indicated essentially complete carboxylation of trenonacog alfa (>11.7 mol Gla/mol FIX; 87% Gla-12 and 13% Gla-10 + Gla-11 by strong anion exchange chromatography), consistent with reported data for nonacog alfa (11.5 mol Gla/mol FIX) [4]. The change in structure due to calcium ion binding of the Gla domain was demonstrated by the decrease in hydrodynamic volume measured by gel filtration of FIX in the presence of calcium (Figure 2(b)).

A second important calcium ion binding site in FIX is found in the junction between the Gla domain and the first epidermal growth factor like (EGF) domain [23, 36]. Aspartic acid residue 64 is part of this calcium ion binding site. In plasma-derived FIX, slightly fewer than 50% of FIX molecules have been modified at this residue by beta-hydroxylation. Trenonacog alfa had measured beta-hydroxylation levels at least equivalent to the plasma-derived and consistent with reported data for nonacog alfa [4].

A number of the posttranslational modification sites are located in the activation peptide of FIX. Among those seen in plasma-derived FIX are sulfation at residue 155 (tyrosine) and phosphorylation at residue 158 (serine). Trenonacog alfa, like other recombinant FIX preparations, showed low levels of modification at these sites [37]. The role of these modifications in FIX function is not clear. No point mutations at either of these residues have been reported in the FIX mutation database [38]. When comparing the pharmacokinetic properties of the first recombinant FIX product, nonacog alfa, against plasma-derived, it was noted that recombinant FIX had a lower* in vivo* recovery [39, 40]. It was suggested that the reduced levels of sulfation and phosphorylation in recombinant FIX might be responsible for the lower recovery of that product [39]. Interestingly, all recombinant FIX products have low levels of sulfation and phosphorylation, but there is no consistent correlation with pharmacokinetics of plasma-derived FIX, suggesting that sulfation and phosphorylation do not play a critical role in determining pharmacokinetic parameters [39, 41].

By contrast, glycosylation strongly affects recovery and half-life of many proteins including FIX [41–43]. In FIX, there are two potential N-linked and four potential O-linked glycosylation sites in the activation peptide, and two potential O-linked glycosylation sites in the EGF domain. The N-glycans of trenonacog alfa are of the complex classification, highly branched, contain poly-N-acetyllactosamine repeats, and are highly sialylated. These structural features are similar to those found in plasma-derived FIX [10]. The O-glycans in the EGF domain and the activation peptide of trenonacog alfa are identical to those found in the corresponding sites of plasma-derived factor IX and other recombinant factor IX products [7, 8, 21]. In FIX, terminal sialic acid content is important for the pharmacokinetics of FIX and removal of the sialic acids dramatically reduces recovery and half-life of FIX [44]. Consistent with these findings, a pharmacokinetic study comparing trenonacog with nonacog alfa, which did not show a statistically significant difference between the two recombinant products, did show that area under the curve and other pharmacokinetic values correlate with the degree of sialylation of the N-glycans [41]. In general, this suggests that higher levels of sialylation should be associated with better pharmacokinetics [41–43].

To be functional* in vivo*, FIX should be activated and the activated form should bind to factor VIIIa to cleave factor X. Activation of FIX was monitored on gels by the appearance of heavy and light chain. The rate of appearance of activation products in trenonacog alfa was consistent across all lots. Titrating FIXa with a known concentration of antithrombin indicated that greater than 90% of FIX was activated in all of the trenonacog alfa lots examined. FIXa activity was assessed as the ability of FVIIIa to accelerate FX activation. The acceleration was consistent across all lots and reflective of tight binding of the activated trenonacog alfa product to FVIIIa with good activity toward FX [35].

The specific activity is a combined assessment of activation, activity, and regulation by antithrombin. Specific activity is determined from a clotting assay done in contact-activated plasma and represents a comparison to native FIX in normal pooled plasma. By definition, a protein (FIX) present at 4 *μ*g/mL in normal plasma has a maximum specific activity of 250 IU/mg. The trenonacog alfa lots that were evaluated had a mean specific activity of 247 IU/mg with minimal lot-to-lot variation (RSD = 7.15%). This value is consistent with the reported specific activity of nonacog alfa (260 IU/mg) [4]. These specific-activity data support the fact that trenonacog alfa performs in a manner that is consistent with a fully functional, high quality FIX product.

## 5. Conclusions

The results indicate that IXINITY (trenonacog alfa [coagulation factor IX (recombinant)]) is a highly pure product with a primary sequence and posttranslational modifications consistent with the common Thr-148 polymorphism of plasma-derived FIX.* In vitro* testing verified that trenonacog alfa is fully functional in the coagulation cascade and that this functionality is highly consistent lot-to-lot.

## Figures and Tables

**Table 1:** Posttranslational modifications in trenonacog alfa, nonacog alfa, and plasma-derived factor IX.

Posttranslational modification	Trenonacog alfa	Nonacog alfa	Plasma-derived factor IX
Disulfide bridging	Eleven disulfide bridges	Eleven disulfide bridges	Eleven disulfide bridges [4]

*γ*-Carboxylation	≥11.7 mol *γ*-carboxyglutamic acid/mol factor IX	11.5 mol *γ*-carboxyglutamic acid/mol factor IX [4]	12 mol *γ*-carboxyglutamic acid/mol factor IX [5]

*β*-Hydroxylation	50% *β*-hydroxylated at Asp64	46% *β*-hydroxylated at Asp64	~40% *β*-hydroxylated at Asp64 [4]

Sulfation	Tyr155: low degree of sulfation observed (5%)	Tyr155: low degree of sulfation observed	Tyr155: high degree of sulfation observed [4]

Phosphorylation	Ser158: low degree of phosphorylation observed (3%)	Ser158: low degree of phosphorylation observed	Ser158: ~90% phosphorylated [6]

O-linked glycosylation			
EGF domain	Ser53: Xyl_1,2_-Glc Ser61: Neu5Ac-Hex-HexNAc-Fuc	Ser53: Xyl_1,2_-Glc [21] Ser61: Neu5Ac-Gal-GlcNAc-Fuc [21]	Ser53: Xyl_1,2_-Glc [7] Ser61: Neu5Ac-Gal-GlcNAc-Fuc [7, 8]
Activation peptide	Low degree of site occupancy observed (13%)	Low degree of site occupancy observed [4]	Low degree of site occupancy observed [9]

N-linked glycosylation	N-glycans are complex, predominantly tri- and tetraantennary, and predominantly sialylated; 80% of N-glycans are either tetrasialylated or trisialylated	N-glycans are complex, predominantly tri- and tetraantennary, and predominantly sialylated [4]	N-glycans are complex, predominantly tri- and tetraantennary, and predominantly sialylated [10]

Total sialylation	8.8 mol sialic acid/mol factor IX	6.5 mol sialic acid/mol factor IX [9]	8.8 mol sialic acid/mol factor IX [9]

**Table 2:** Functional characterization of trenonacog alfa.

	Lot 1	Lot 2	Lot 3	Lot 4	Lot 5	Lot 6	Mean	% RSD
Specific activity (IU/mg)	216	256	247	269	249	242	247	7.15
FIX activation rate (fraction FIXa band intensity^−1^·minute^−1^)	0.0182	0.0199	0.0157	0.0185	0.0171	0.0137	0.0172	12.9
Rate of increase in FIXa activity in presence of FVIIIa relative to absence of FVIIIa	1.79 × 10^3^	1.85 × 10^3^	1.75 × 10^3^	1.65 × 10^3^	1.90 × 10^3^	1.82 × 10^3^	1.79 × 10^3^	4.86
Titration with AT; slope of inhibition curve (mIU FIXa·mL^−1^·*μ*M AT^−1^)	−1.29 × 10^4^	−1.53 × 10^4^	−1.44 × 10^4^	−1.81 × 10^4^	−1.42 × 10^4^	−1.44 × 10^4^	−1.49 × 10^4^	11.7

## Trademark Legend

IXINITY and any and all Emergent BioSolutions Inc. brands, products, service and feature names, logos, and slogans are trademarks or registered trademarks of Emergent BioSolutions Inc. or its subsidiaries in the United States or other countries. All rights are reserved.
